# Association of Ultrasound-Derived Metrics of the Quadriceps Muscle with Protein Energy Wasting in Hemodialysis Patients: A Multicenter Cross-Sectional Study

**DOI:** 10.3390/nu12113597

**Published:** 2020-11-23

**Authors:** Sharmela Sahathevan, Ban-Hock Khor, Birinder Kaur Sadu Singh, Alice Sabatino, Enrico Fiaccadori, Zulfitri Azuan Mat Daud, Mohammad Syafiq Ali, Sreelakshmi Sankara Narayanan, Dina Tallman, Karuthan Chinna, Bak-Leong Goh, Abdul Halim Abdul Gafor, Ghazali Ahmad, Zaki Morad, Pramod Khosla, Tilakavati Karupaiah

**Affiliations:** 1 Dietetics Program, Faculty of Health Sciences, Universiti Kebangsaan Malaysia, Jalan Raja Muda Abdul Aziz, Kuala Lumpur 50300, Malaysia; sham_0901@yahoo.com; 2 Department of Medicine, Faculty of Medicine, Universiti Kebangsaan Malaysia, Jalan Yaakob Latif, Bandar Tun Razak, Cheras, Kuala Lumpur 56000, Malaysia; khorbanhock@gmail.com (B.-H.K.); halimgafor@gmail.com (A.H.A.G.); 3 Nutrition Program, Faculty of Health Sciences, Universiti Kebangsaan Malaysia, Jalan Raja Muda Abdul Aziz, Kuala Lumpur 50300, Malaysia; birin@ppukm.ukm.edu.my; 4 Nephrology Unit, Department of Medicine and Surgery, Parma University Hospital, 43121 Parma, Italy; alice.sabatino86@gmail.com (A.S.); enrico.fiaccadori@unipr.it (E.F.); 5 Department of Dietetics, Faculty of Medicine and Health Sciences, Universiti Putra Malaysia, UPM Serdang 43400, Selangor, Malaysia; zulfitri@upm.edu.my (Z.A.M.D.); mohammadsyafiqali14@gmail.com (M.S.A.); 6 School of BioSciences, Faculty of Health and Medical Sciences, Taylor’s University Lakeside Campus, No 1, Jalan Taylor’s, Subang Jaya 47500, Selangor, Malaysia; sreelakshmi.narayanan@taylors.edu.my; 7 Department of Nutrition and Food Science, Wayne State University, Detroit, MI 48202, USA; dina.tallman@wayne.edu (D.T.); aa0987@wayne.edu (P.K.); 8 School of Medicine, Faculty of Health and Medical Sciences, Taylor’s University Lakeside Campus, No 1, Jalan Taylor’s, Subang Jaya 47500, Selangor, Malaysia; karuthan@gmail.com; 9 Department of Nephrology, Serdang Hospital, Jalan Puchong, Kajang 43000, Selangor, Malaysia; bakleong@gmail.com; 10 Department of Nephrology, Kuala Lumpur Hospital, Jalan Pahang, Wilayah Persekutuan Kuala Lumpur 50856, Malaysia; ghazaliahmad2018@gmail.com; 11 Department of Cardiology, National Heart Institute, 145, Jalan Tun Razak, Kuala Lumpur 50400, Malaysia; 12 National Kidney Foundation, 70, Jalan 14/29, Petaling Jaya 46100, Selangor, Malaysia; zakimorad@gmail.com

**Keywords:** ultrasound imaging, hemodialysis, protein energy wasting, muscle wasting, quadriceps muscle

## Abstract

This study aimed to assess muscle wasting and risk of protein energy wasting (PEW) in hemodialysis (HD) patients using an ultrasound (US) imaging method. PEW was identified using the ISRNM criteria in 351 HD patients. Quadriceps muscle thickness of *rectus femoris* (RF) and *vastus intermedius* (VI) muscles and cross-sectional area (CSA) of the RF muscle (RF_CSA_) were measured using US and compared with other physical measures. Associations of US indices with PEW were determined by logistic regression. Irrespective of gender, PEW vs. non-PEW patients had smaller RF, VI muscles, and RF_CSA_ (all *p* < 0.001). US muscle sites (all *p* < 0.001) discriminated PEW from non-PEW patients, but the RF_CSA_ compared to bio-impedance spectroscopy had a greater area under the curve (AUC, 0.686 vs. 0.581), sensitivity (72.8% vs. 65.8%), and specificity (55.6% vs. 53.9%). AUC of the RF_CSA_ was greatest for PEW risk in men (0.74, 95% CI: 0.66–0.82) and women (0.80, 95% CI: 0.70–0.90) (both *p* < 0.001). Gender-specific RF_CSA_ values (men < 6.00 cm^2^; women < 4.47 cm^2^) indicated HD patients with smaller RF_CSA_ were 8 times more likely to have PEW (AOR = 8.63, 95% CI: 4.80–15.50, *p* < 0.001). The US approach enabled discrimination of muscle wasting in HD patients with PEW. The RF_CSA_ was identified as the best US site with gender-specific RF_CSA_ values to associate with PEW risk, suggesting potential diagnostic criteria for muscle wasting.

## 1. Introduction

Muscle depletion is a clinical feature of protein energy wasting (PEW), a malnutrition state coexisting with inflammation and cachexia in chronic kidney disease (CKD) patients [1]. Currently, PEW is identified when three out of four diagnostic criteria recommended by the International Society of Renal Nutrition and Metabolism (ISRNM) expert panel are met, namely, (i) biochemical criteria, (ii) low body weight, reduced total body fat, or weight loss, (iii) decreased muscle mass, and (iv) reduced protein and energy intakes [1]. Global prevalence of PEW in hemodialysis (HD) patients is estimated at 28% to 54% [2]. Muscle wasting aggravates existing comorbidities, resulting in increased risk of morbidity and mortality [3,4]. Low muscle mass is also associated with reduced strength and mobility leading to frailty, low quality of life, and increased risk of hospitalization and death afflicting patients at the later stages of CKD [4,5].

Non-invasive methodologies assessing muscle wasting allow for timely treatment, but existing methods have disadvantages in terms of accuracy, cost, and feasibility [3,5]. The mid-arm muscle circumference (MAMC), a proxy single-site upper limb anthropometric measure, is based on a predictive equation estimating whole-body muscle mass [5,6,7], but is highly prone to technical errors of measurement [6]. Bio-impedance analysis (BIA) and bio-impedance spectroscopy (BIS) enable muscle mass quantification, but is hindered by the overhydration status typical to HD patients [5]. Additionally, these methods are still proxy measures of the lean body mass (LBM) relying on predictions based on the hydration status to estimate muscle mass [6,7,8]. Gold standard imaging techniques such as magnetic resonance imaging, computed tomography (CT), or dual energy X-ray absorptiometry have high accuracy and reliability, but are cost-prohibitive, require specialized interpretive skills, and risk radiation exposure [5]. We previously validated the US method by measuring quadriceps muscle thickness (QMT) and cross-sectional area (CSA) of the mid-thigh region against the CT scan, which suggested that US is a simple, accurate, and clinically applicable approach for muscle wasting assessment in HD patients [9], which was not impacted by the overhydration status [10,11].

The present study therefore evaluated the US method to identify HD patients at risk of PEW by targeting measures of QMT and CSA. The quadriceps muscle, due to its large size and assessment ease [12], was the preferred site for measurement, as lower limbs are more susceptible to uremic myopathy, and QMT deterioration can be easily gauged [13,14]. We also evaluated US measures in relation to (i) differences between nutritional assessment parameters in PEW and non-PEW patients, (ii) comparisons with physical parameters in identifying PEW risk, (iii) gender-specific US value for PEW identification, and (iv) association of the US value with PEW risk.

## 2. Materials and Methods

### 2.1. Study Design and Patient Recruitment

**Figure 1:**
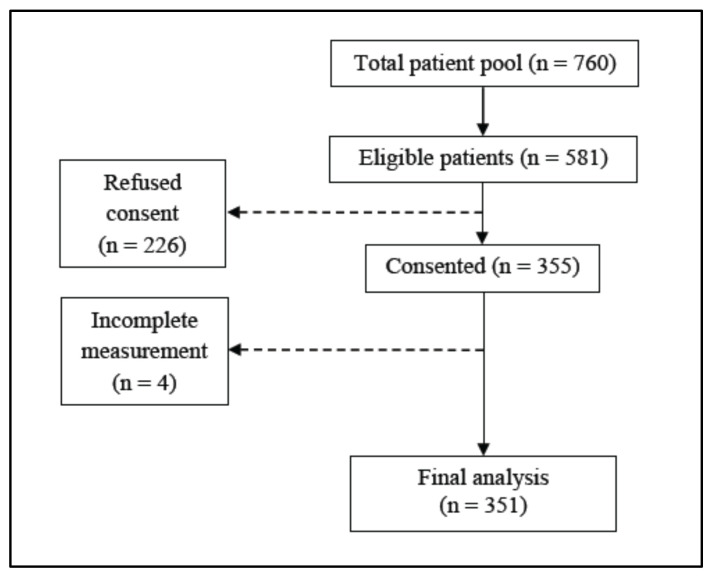
Study flow of patients for recruitment.

This cross-sectional study was undertaken with maintenance HD patients screened for the Palm Tocotrienol in Chronic Hemodialysis (PATCH) study in Malaysia. Patient recruitment was conducted between September 2015 and September 2016 at four public hospitals and six non-governmental dialysis centers in the Klang Valley where patients access 4-hourly dialysis sessions three times weekly as per the standard protocol. Eligibility criteria were patients dialyzing for ≥ 3 months, aged ≥ 18 years and who had provided written consent. Patients with repeated history of hospitalizations, inter-current illnesses over six months prior to the recruitment, or diagnosed with inflammatory diseases or malignancy were excluded. The stock flow of patients included in the final analysis is presented in Figure 1.

The study was approved by the Medical Research and Ethics Committee, Ministry of Health, Malaysia (NMRR-15-865-25260) and the Medical Research Ethics Committee of the National University of Malaysia (NN-039-2015).

### 2.2. Ultrasound Measures

**Figure 2:**
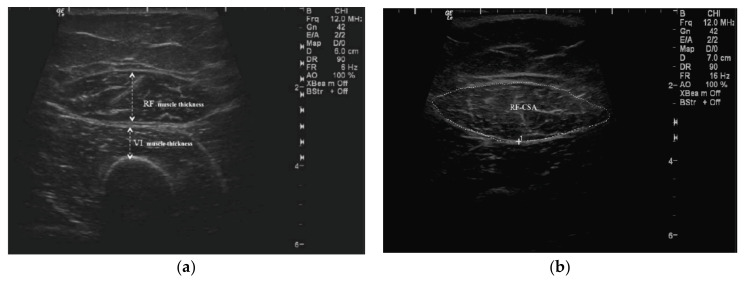
Quadriceps muscle thickness (QMT) and cross-sectional area (CSA): (**a**) QMT of *rectus femoris* (RF) and *vastus intermedius* (VI) muscles; (**b**) RF_CSA._

QMT and CSA were assessed using a portable US device (GE Logiq e Digital Portable Color Doppler, GE Healthcare, Wauwatosa, US). Standardized anatomical landmarking was performed as per the International Society for the Advancement of Kinanthropometry (ISAK) protocol [6] by an ISAK-trained anthropometrist (T.K.) as detailed previously [9]. Researchers (S.S. and B.-H.K.) performed the US scan two hours after dialysis commencement, with patients’ dialysis chairs adjusted for the supine position and both knees extended but relaxed. QMT of *rectus femoris* (RF) and *vastus intermedius* (VI) muscles were determined (Figure 2a) on both mid- (MID) and 2/3-length landmarks on both thighs. CSA of the RF muscle (RF_CSA_) was measured at the mid-thigh landmark (Figure 2b). Scans were performed on both thighs, and the data reported were average of the left and right measurements. Two readings were obtained for each measured site, and the mean value was used for data analysis.

#### 2.2.1. QMT Assessment

Firstly, the scanning depth of the US transducer was adjusted to view the femur at the designated points, the mid-thigh and 2/3-length, and positioned perpendicular to the long axis of the thigh. The transducer was then moved along the axis of the thigh to identify the thickest width of the RF muscle with the VI and femur visualised in a single field. The identified site was centered on the femur to reflect this view and this image was captured. The QMT was drawn as “inner-to-inner” septa of the thickest region of the RF muscle, whereas the highest point from the femur to the outer septa of the RF represented muscle thickness for the VI.

#### 2.2.2. RF_CSA_ Measurement

The same protocol for QMT thickness was applied for the RF_CSA_ measurement. In cases of patients with larger muscles, the panoramic view option was used to maneuver the transducer smoothly from left to right along the axis of the identified site to capture the image of the muscle septa. The inner echogenic line of the RF muscle was outlined by a moving cursor before freezing the image to calculate the RF_CSA_ in cm^2^.

### 2.3. Nutritional Status Assessment

#### 2.3.1. Anthropometry

Anthropometric data were collected by the same dietitian (S.S.) to eliminate inter-observer variation. These included post-dialysis weight (SECA Model 220, SECA, Germany), body mass index (BMI), triceps skinfold (TSF) thickness as per the ISAK protocol on the dominant or non-fistula arm with a Harpenden skinfold caliper (HSK-BI, British Indicators, West Sussex, UK) [6], and mid-arm circumference (MAC) with a non-stretchable tape (Lufkin^®^, Apex Tool Group, LLC, NC, USA). MAMC and mid-arm muscle area (MAMA) were calculated using the Heymsfield equations [15]. The mid-thigh girth measurement was also taken at the mid-point of both legs as per the ISAK protocol [6].

#### 2.3.2. Body Composition

Body composition was assessed using a portable whole-body BIS device (Body Composition Monitor, Fresenius Medical Care, Bad Homburg, Germany) with the patient resting in the supine position before dialysis. The hydration status, lean tissue mass (LTM), and fat tissue mass (FTM) data generated were based on the physiologic tissue model [8]. Both lean tissue index (LTI) and fat tissue index (FTI) are indices of LTM and FTM corrected for patients’ heights. Body cell mass (BCM) includes both visceral and somatic protein stores without inclusion of extracellular fluids and bone minerals. Similarly, adipose tissue mass (ATM) is the fat tissue mass without inclusion of extracellular fluids and bone minerals.

#### 2.3.3. Laboratory Investigations

Routine biochemistry parameters for serum urea, creatinine, and total iron-binding capacity were based on in-house laboratory analyses as per the standard operating procedures accredited by the Ministry of Health, Malaysia. Additional tests, such as serum albumin (bromocresol green method), high-sensitivity C-reactive protein (hsCRP) (particle-enhanced immunoturbidimetric assay), and bicarbonate (enzymatic method) were analyzed by an accredited external laboratory. Interleukin-6 (IL-6) measurement using an enzyme-linked immunosorbent assay was performed at an institutional laboratory (S.S.N. and B.-H.K.). All biochemistry analyses were based on mid-week-collected fasting blood samples.

#### 2.3.4. Dietary Assessment

24-hour dietary records were collected for three days, inclusive of two random weekdays and one optional weekend day [16]. Energy and protein intakes were analyzed using Nutritionist Pro^TM^ 2.2.16 (First DataBank Inc., San Bruno, CA, USA, 2004).

#### 2.3.5. Nutritional Risk Assessment

The malnutrition–inflammation score (MIS) rating assessed the severity of effect of the malnutrition–inflammation complex syndrome on the nutritional status [17]. The cumulative score for the MIS ranges between 0 (normal) and 30 (severely malnourished).

#### 2.3.6. Handgrip Strength Test

Handgrip strength (HGS) was assessed using a handgrip dynamometer (Jamar^®^, BK-7498; Fred Sammons, Inc., Burr Ridge, IL, USA) on the dominant or non-fistula hand [18]. The median of three readings was used as the final result.

#### 2.3.7. Protein Energy Wasting Assessment

PEW was diagnosed according to these ISRNM diagnostic criteria [1]: BMI < 23 kg/m^2^, reduction > 10% in MAMC in relation to the 50th percentile of the reference population, serum albumin < 38 g/dL, and dietary energy intake (DEI) < 25 kcal/kg ideal body weight (IBW).

### 2.4. Statistical Analysis

Variables were presented as mean ± standard deviation (SD), median (interquartile range (IQR)), or frequency (percentage). The normal distribution of continuous variables was assessed using the Kolmogorov–Smirnov test. Group differences were analyzed using the Student’s *t*-test and the Mann–Whitney’s U-test for normally and non-normally distributed data, respectively. Categorical data were analyzed using the Chi-squared test. The receiver operating characteristic (ROC) curve analysis was applied to US measures and physical parameters whereby the area under the curve (AUC) indicated the probability of measures to identify patients at risk of PEW. Cut-off points to discriminate the risk of PEW depended on the highest sensitivity (1-specificity) value from the ROC curve. The logistic regression analysis determined associations of these values with PEW risk and the odds ratio was adjusted for confounding variables (age, ethnicity, comorbidities, and dialysis vintage). All the analyses were computed using the IBM Statistical Package for Social Sciences version 26.0 (IBM SPSS Statistics Inc., Chicago, IL, USA). Statistical significance was set at *p* < 0.05 for all the tested parameters.

## 3. Results

### 3.1. Patient Characteristics as per PEW Identification

Prevalence of PEW was 23.1% (*n* = 81/351). Age, ethnicity, dialysis vintage, and dialysis adequacy did not significantly differ between PEW and non-PEW patients except for gender (*p* = 0.008) (Table 1). PEW compared to non-PEW patients had lower serum urea (*p* < 0.001), creatinine (*p* = 0.001), and albumin (*p* < 0.001), but higher serum bicarbonate (*p* = 0.009). IL-6 and hsCRP were not significantly different between groups. PEW compared to non-PEW patients had lower DEI (21.34 vs. 25.80 kcal/kg IBW, *p* < 0.001) and dietary protein intake (0.81 vs. 0.93 g/kg IBW, *p* = 0.002). MIS values were higher in PEW patients (9 vs. 5, *p* < 0.001) compared to non-PEW patients.

### 3.2. Nutritional Status Assessment and US Measures

Weight, BMI, MAC, TSF, MAMC, MAMA, and mid-thigh girth were all significantly lower (*p* < 0.001) for PEW compared to non-PEW patients irrespective of gender (Table 2).

Body composition measures reflective of the muscle mass, LTM and BCM, did not significantly differ between groups. However, men with PEW compared to men without PEW had lower LTM (34.05 vs. 38.00 kg, *p* = 0.001) and BCM (18.20 vs. 21.20 kg, *p* < 0.001). Irrespective of gender, FTM was significantly lower in PEW patients (13.50 vs. 22.00 kg, *p* < 0.001). After the LTM and FTM were corrected for height, significantly lower LTI (PEW = 11.80 kg/m^2^ vs. non-PEW = 12.90 kg/m^2^, *p* = 0.029) and FTI (PEW = 7.30 kg/m^2^ vs. non-PEW = 12.10 kg/m^2^, *p* < 0.001) were observed in the PEW patients overall; and men with PEW (LTI = 12.35 kg/m^2^ and 14.10 kg/m^2^ in PEW and non-PEW patients, respectively; FTI = 7.95 kg/m^2^ and 11.00 kg/m^2^ in PEW and non-PEW patients, respectively; both *p* < 0.001). The FTI in women was affected by PEW (PEW = 7.00 kg/m^2^ vs. non-PEW = 14.10 kg/m^2^, *p* < 0.001), but not the LTI. Only the HGS of men was significantly affected by the presence of PEW (PEW = 19.50 kg vs. non-PEW = 23.10 kg, *p* = 0.008).

All US measures were significantly lower in PEW compared to non-PEW patients irrespective of gender (*p* < 0.001).

### 3.3. Assessment of US, BIS, and HGS Methods for PEW Risk

Comparison of US measures with BIS-derived muscle measures and the HGS for risk of PEW was determined using the ROC analysis with derived AUC data presented in Table 3. Each US muscle site significantly discriminated PEW from non-PEW patients (all *p* < 0.001). Other measures failed to discriminate PEW from non-PEW patients (all *p* > 0.05) except for BIS−LTI (*p* = 0.029). However, the AUC sensitivity and specificity for BIS−LTI were lower compared to the RF_CSA._

### 3.4. Development of the Gender-Specific Value of US Measures for PEW Risk

**Figure 3:**
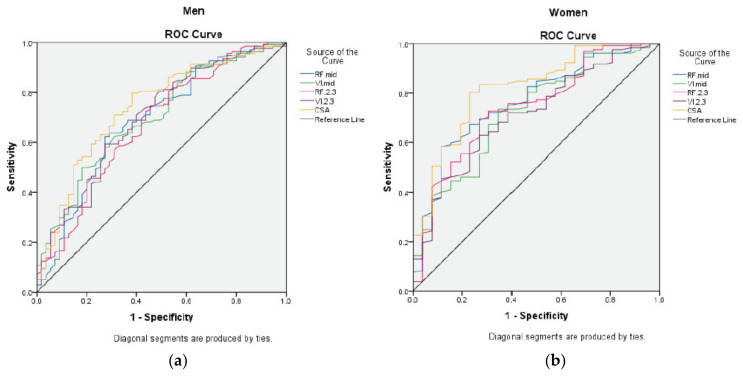
ROC analysis for US measures according to gender. Figure 3 represents the area under curve for risk of PEW according to US muscle sites for (**a**) men; (**b**) women. Abbreviations: CSA, cross-sectional area; MID, mid-point; PEW, protein energy wasting; ROC, receiver operating characteristic; RF, *rectus femoris*; US, ultrasound; VI, *vastus intermedius*.

We next compared the AUC computed for each US measure against the PEW risk for both men and women (Figure 3).

Only the RF_CSA_ measurement elicited the greatest AUC specific to gender (men = 0.74, 95% CI: 0.66–0.82, *p* < 0.001; women = 0.80, 95% CI: 0.70–0.90, *p* < 0.001) compared to other muscle sites (Table 4). Based on the ROC analysis, the best RF_CSA_ cut-off point for men was < 6.00 cm^2^ (sensitivity = 62%, specificity = 80%) and < 4.47 cm^2^ for women (sensitivity = 77%, specificity = 80%) (Table 5). Association of the RF_CSA_ gender-specific values with PEW indicated that HD patients with the RF_CSA_ below these values were 8 times more likely to have PEW (OR = 8.00, 95% CI: 4.62–13.86, *p* < 0.001) compared to those above these values (Table 6). The association remained significant (AOR = 8.63, 95% CI: 4.80–15.50, *p* < 0.001) even after adjustment for confounding factors (Table 6).

## 4. Discussion

PEW, common in maintenance HD patients, greatly contributes towards morbidity and increases mortality risk. We observed for this middle-aged HD population that the prevalence of PEW was 23.1% (*n* = 81) when applying the ISRNM PEW diagnostic criteria [1]. Low cost and direct clinical assessment of muscle wasting would aid diagnosis of at-risk patients. Our study demonstrated the ability of a US method to directly discriminate HD patients with PEW as per muscle thickness and CSA of the quadriceps muscle. We further showed that this US assessment also discriminated PEW risk according to gender.

To our knowledge, this is the first study to identify the associations of US measurements with PEW in HD patients. As expected, the ISRNM PEW diagnostic criteria successfully revealed PEW patients having worse nutritional indices related to anthropometry, muscle mass and physical strength, dietary intake and the MIS score. The ISRNM PEW criteria use objective markers with specific values to indicate the risk of malnutrition [1]. Importantly, this method includes quantification of a patient’s actual dietary intake [19] given that a suboptimal dietary intake is an underlying cause of PEW [1].

Our primary objective was to assess muscle wasting using a US procedure in an HD population discriminated by their PEW diagnosis. The non-PEW patients served as a “normal” comparator in this study. Consistent with our utilization of this US method, Sabatino et al. (2019) observed significantly smaller QMT, namely of the RF and VI muscles, in malnourished HD patients (MIS ≥ 6), as well as a significant negative correlation between MIS and US muscle sites [11]. However, assessing the ability of the US method to discriminate PEW risk according to the ISRNM diagnostic criteria was not the intention of this study [11]. Our study clearly indicated that along with QMT, the additional mid-thigh CSA of the RF muscle was significantly lower in PEW compared to non-PEW patients.

Contrarily, applying BIS and HGS could only differentiate muscle wasting in men with PEW. This may be because men compared to women are more vulnerable to muscle wasting [18,20]. This observation relates to men having more muscle mass whilst women have more fat mass [21], suggesting that gender differences for body composition should be factored in when interpreting muscle measures [5]. In contrast, US measures were able to distinguish muscle wasting irrespective of gender in PEW compared to non-PEW patients.

Importantly, our study allowed for the generation of clinically meaningful RF_CSA_ gender-specific values to facilitate screening and identification of HD patients at risk of PEW. We found the PEW risk to be 8 times higher in the HD patients with the RF_CSA_ lower than gender-specific values for men (<6.00 cm^2^: AUC = 0.74, sensitivity = 62%, specificity = 80%) and women (<4.47 cm^2^: AUC = 0.80, sensitivity = 77%, specificity = 80%). For the purpose of sarcopenia diagnosis in the elderly (73.7 ± 9.2 years) pre-dialysis CKD population, Souza et al. (2018) also identified the RF_CSA_ gender-specific values (men < 13.25 mm^2^; women < 10.95 mm^2^) using the US approach for muscle wasting [22]. Of note, the definition of sarcopenia is different from the PEW diagnosis, as it is only characterized by low muscle mass and muscle strength [23]. Dialysis compared to pre-dialysis CKD patients are more prone to muscle wasting as the HD procedure itself is an iatrogenic factor of malnutrition promoting muscle proteolysis [24]. As regards to sarcopenia, the *Asian Working Group for Sarcopenia* has noted lower limits for low muscle mass in Asians compared to the Western population due to gender, body size, and ethnic differences [25], thus emphasizing the need to generate country-specific values applicable to stage 5 CKD patients, allowing for more meaningful interpretation of local data.

The RF_CSA_ was the most superior site amongst the examined US measures in discriminating PEW risk compared to other physical parameters for body composition. Other studies have reported significant correlations between the RF_CSA_ and the CT-derived LBM for upper (*r* = 0.286) and lower limbs (*r* = 0.271), BIA-derived whole-body fat-free mass (*r* = 0.430), HGS (*r* = 0.300), and physical performance measures in pre-dialysis CKD patients [22,26] and chronic obstructive pulmonary disease patients [27]. While the quadriceps comprises four muscle groups [27] and the RF_CSA_ only constitutes 10% of the total quadriceps muscle CSA [28], this single site’s US measure when applied to sarcopenia diagnosis was a significant predictor of post-discharge disposition and duration of hospitalization [29]. Thus, association of the RF_CSA_ observed in our study suggests that this US site has great utility as a surrogate measure for whole-body muscle mass and physical strength.

Besides being a large skeletal muscle group in the body enabling easy assessment [12], the strong positive correlation between muscle measures and the strength of quadriceps muscles [14] is indicative of the appropriateness of the quadriceps site to assess muscle wasting. The quadriceps isometric maximum voluntary contraction produced by the knee extensor muscles correlates to the QMT and RF_CSA_ and deterioration of the quadriceps would therefore affect physical function which is also observed in PEW patients [26]. Several studies have also investigated muscle wasting of the quadriceps muscle in HD [9,11,30], pre-dialysis CKD [22], critically ill [13,31], and chronic obstructive pulmonary disease patients [27]. Losses and gains in the quadriceps muscle may be monitored when evaluating intervention based on exercise [14,32] or a combination of nutritional supplementation and exercise [33], indicating that this site is also suitable for detecting changes in response to rehabilitation.

The immediate strength of the current study is that our observations are based on the largest multicenter sampling performed for US assessment of muscle wasting involving HD patients from both public and non-governmental organization dialysis centers. This large dataset enabled generation of the RF_CSA_ gender-specific values for identification of patients at risk of PEW. However, application of these values for PEW risk to HD populations in other countries warrants further investigation. US scans in this study were completed within 15 min without hindrance to the patient’s dialysis schedule, thereby emphasizing convenience, feasibility, and non-invasive nature of the technique for assessment of muscle wasting and PEW risk.

As for the practical issues related to US imaging, the measurement is operator-dependent [14], but this limitation is overcome with adequate training, followed by optimization and adherence to the protocol [10]. Health practitioners with minimal training of two weeks could perform this US procedure [14,27]. Recently, Bury et al. (2020) documented successful performance of US scans by dietitians following appropriate training [31]. Our assessors adhered to the US protocol by avoiding oblique images or additional pressure while scanning which may cause muscle compression [10,14], as per the training provided by an experienced team (A.S. and E.F.). Importantly, landmarking of sites for US scanning was performed as per the ISAK protocol, which reduced the technical errors of measurement [6]. As for operator dependency, good intra- and inter-reliability of US measures have been previously reported [9,34].

Limitations inherent to cross-sectional studies are also acknowledged in this study, as cause–effect relationship or prognostic ability of the RF_CSA_ values in predicting hospitalization or mortality risk for PEW patients could not be investigated. With four obese patients in our study, we faced difficulty in locating the femur and poor visualization of RF muscle borders. Therefore, generalization of these results to obese patients will be restricted. Further, exclusion of patients with amputation and reduced mobility may have led to selection bias, as these patients experience greater muscle wasting due to their physical inability. Information on the physical activity level, a factor that may influence muscle status, was unavailable, as it was not part of the data collected for the screening study.

## 5. Conclusions

Our study indicated that the US method discriminated muscle wasting in HD patients with PEW irrespective of gender. The RF_CSA_, a single site measure of the lower limbs, was a better indicator of PEW risk compared to other indirect approaches. The RF_CSA_ was identified as the best US site and the developed gender-specific RF_CSA_ values were associated with PEW risk. Therefore, application of a US method in a clinical setting should be considered for a rapid screening of PEW risk. Future studies investigating prospective evaluation of the US values for hospitalization and mortality risk in PEW patients as well as evaluating changes in muscle status using a US method as a targeted outcome of nutritional intervention in treating PEW is highly encouraged.

## Figures and Tables

**Table 1:** Patient characteristics as per PEW identification.

Variables *^a,b^*	Overall (*n* = 351)	PEW *^c^* (*n* = 81)	Non-PEW (*n* = 270)	*p*-Value *^d^*
Age (years)	55.18 ± 14.04	52.79 ±15.84	55.89 ± 13.40	0.112
**Gender**				
Male	193 (55.0)	55 (67.9)	138 (51.1)	0.008
Female	158 (45.0)	26 (32.1)	132 (48.9)	
**Ethnicity**				
Malay	102 (29.1)	25 (30.9)	77 (28.5)	0.118
Chinese	190 (54.1)	37 (45.7)	153 (56.7)	
Indian	59 (16.8)	19 (23.5)	40 (14.8)	
**Comorbidities**				
Diabetes	146 (41.6)	24 (29.6)	122 (45.2)	0.013
Hypertension	274 (78.1)	55 (67.9)	219 (81.1)	0.012
CVD	56 (16.0)	11 (13.6)	45 (16.7)	0.506
Dialysis vintage (months)	82 ± 72	85 ± 88	80 ± 67	0.632
Dialysis adequacy (Kt/V)	1.65 (1.40–1.87)	1.71 (1.41–2.04)	1.63 (1.39–1.83)	0.100
**Biochemistry Markers**				
• Serum urea (mmol/L)	19.2 (15.60–22.85)	17.05 (14.38–20.08)	20.15 (16.38–23.40)	<0.001
• Serum creatinine (μmol/L)	814 (672–951)	738 (624–884)	834 (704–976)	0.001
• Serum albumin (g/L)	40 (37–42)	37 (35–42)	40 (38–42)	<0.001
• hsCRP (mg/L)	3.50 (1.60–7.60)	3.48 (1.44–6.45)	3.53 (1.70–7.96)	0.563
• IL–6 (pg/mL)	3.71 (2.33–5.99)	4.43 (2.30–8.90)	3.63 (2.33–5.69)	0.166
• Serum bicarbonate (mmol/L)	20.64 (18.87–22.63)	21.25 (19.88–23.14)	20.53 (18.38–22.42)	0.009
**Dietary Intake**				
• DEI (kcal/kg IBW)	24.37 (20.82–28.85)	21.34 (18.86–23.60)	25.80 (21.97–29.86)	<0.001
• DPI (g/kg IBW)	0.89 (0.71–1.12)	0.81 (0.65–1.02)	0.93 (0.74–1.16)	0.002
MIS	5 (3–8)	9 (5–11)	5 (3–7)	<0.001

Abbreviations: CVD, cardiovascular disease; DEI, dietary energy intake; DPI, dietary protein intake; hsCRP, high-sensitivity C-reactive protein; IBW, ideal body weight; IL-6, interleukin-6; IQR, interquartile range; MIS, malnutrition–inflammation score; PEW, protein energy wasting. *^a^* Data expressed as *n* (%) for the categorical data; mean ± standard deviation (SD) or median (interquartile range) for the continuous data. *^b^* Categorical data were analyzed using the chi-squared test whilst continuous data were analyzed using the Mann–Whitney U-test. *^c^* PEW was diagnosed when any three out of the four ISRNM diagnostic criteria were met [1], namely, BMI < 23 kg/m^2^, reduction > 10% in the MAMC in relation to the 50th percentile of the reference population, serum albumin < 38 g/dL, and dietary energy intake (DEI) < 25 kcal/kg IBW. *^d^* Significance at *p* < 0.05.

**Table 2:** Nutritional assessment parameters as per PEW identification.

	Overall (*n* = 351)		Men (*n* = 193)		Women (*n* = 158)	
Variable *^a^*	PEW (*n* = 81) Median (IQR)	Non-PEW (*n* = 270) Median (IQR)	PEW (*n* = 55) Median (IQR)	Non-PEW (*n* = 138) Median (IQR)	PEW (*n* = 26) Median (IQR)	Non-PEW (*n* = 132) Median (IQR)
*Anthropometrics*						
Weight (kg)	50.60 (44.45–59.35)	63.00 (54.25–71.08) *^b^*	56.00 (49.50–62.70)	67.15 (59.88–74.30) *^b^*	43.05 (38.60–47.18)	57.35 (50.10–65.86) *^b^*
BMI (kg/m^2^)	20.09 (18.51–22.04)	25.08 (22.96–27.90) *^b^*	20.62 (19.12–22.36)	25.06 (23.46–27.50) *^b^*	18.66 (16.98–20.48)	25.09 (22.27–28.56) *^b^*
MAC (cm)	24.55 (22.53–26.58)	30.10 (27.90–32.76) *^b^*	25.25 (23.20–27.65)	30.18 (28.05–32.40) *^b^*	22.58 (21.13–25.89)	30.05 (27.25–33.71) *^b^*
TSF (mm)	11.80 (8.60–15.75)	16.75 (12.98–23.18) *^b^*	11.30 (8.20–14.90)	14.80 (11.45–19.50) *^b^*	12.75 (9.73–17.28)	20.50 (15.55–30.65) *^b^*
MAMC (cm)	20.37 (18.70–22.94)	24.35 (22.34–26.10) *^b^*	22.05 (19.83–23.52)	25.17 (23.71–26.47) *^b^*	18.49 (17.53–19.48)	23.03 (21.32–25.23) *^b^*
MAMA (cm^2^)	23.95 (20.00–31.89)	38.87 (32.07–45.33) *^b^*	28.67 (21.29–34.05)	40.71 (34.79–46.03) *^b^*	20.13 (17.39–23.71)	35.79 (29.84–44.17) *^b^*
Mid-thigh girth (cm)	42.10 (39.70–46.08)	48.90 (45.38–52.45) *^b^*	43.30 (40.70–47.00)	48.75 (45.50–52.21) *^b^*	40.06 (38.38–42.80)	49.00 (45.23–53.05) *^b^*
*Body Composition and Physical Strength Test*						
LTM (kg)	31.30 (27.00–35.10)	31.30 (26.60–38.30)	34.05 (29.20–37.48)	38.00 (33.70–43.60) *^c^*	26.60 (22.40–29.90)	26.85 (23.20–29.70)
FTM (kg)	13.50 (9.70–20.00)	22.00 (17.10–27.10) *^b^*	14.40 (10.00–21.25)	21.10 (15.50–25.10) *^b^*	11.30 (8.35–16.35)	24.05 (18.00–28.65) *^b^*
LTI (kg/m^2^)	11.80 (10.90–13.50)	12.90 (11.10–14.60) *^c^*	12.35 (11.15–14.43)	14.10 (12.90–16.50) *^b^*	11.40 (10.50–12.20)	11.45 (10.10–12.90)
FTI (kg/m^2^)	7.30 (5.50–10.00)	12.10 (9.20–15.60) *^b^*	7.95 (5.33–10.53)	11.00 (8.20–12.90) *^b^*	7.00 (5.55–9.45)	14.10 (10.73–16.88) *^b^*
ATM (kg)	18.30 (13.20–27.30)	30.00 (23.20–36.80) *^b^*	19.60 (13.60–28.98)	28.70 (21.10–34.20) *^b^*	15.40 (11.45–22.20)	32.70 (24.43–39.03) *^b^*
BCM (kg)	16.50 (14.00–19.30)	17.30 (14.10–21.60)	18.20 (14.88–21.43)	21.20 (18.30–25.40) *^b^*	13.30 (11.55–15.60)	14.50 (11.75–16.48)
HGS (kg)	17.50 (13.40–22.70)	18.30 (14.05–24.60)	19.50 (15.60–26.00)	23.10 (18.25–28.95) *^c^*	13.85 (10.23–16.93)	14.95 (12.10–18.10)
*US Measures*						
RF_MID_ (cm)	1.55 (1.32–1.86)	1.77 (1.55–2.03) *^b^*	1.74 (1.48–1.97)	1.97 (1.74–2.19) *^b^*	1.33 (1.13–1.49)	1.59 (1.41–1.77) *^b^*
VI_MID_ (cm)	1.26 (0.95–1.67)	1.65 (1.29–2.10) *^b^*	1.45 (0.97–1.65)	1.71 (1.39–2.15) *^b^*	1.16 (0.90–1.69)	1.61 (1.22–2.05) *^b^*
RF_2/3_ (cm)	1.20 (0.98–1.48)	1.42 (1.16–1.63) *^b^*	1.31 (1.09–1.60)	1.55 (1.30–1.76) *^b^*	1.01 (0.79–1.19)	1.27 (1.03–1.46) *^b^*
VI_2/3_ (cm)	0.98 (0.75–1.25)	1.27 (0.99–1.63) *^b^*	1.01 (0.76–1.34)	1.28 (1.05–1.63) *^b^*	0.94 (0.71–1.20)	1.22 (0.91–1.64) *^b^*
RF_CSA_ (cm^2^)	5.21 (4.10–6.21)	6.27 (5.09–7.44) *^b^*	5.81 (5.08–6.68)	7.18 (6.07–8.25) *^b^*	4.06 (3.11–4.56)	5.52 (4.61–6.32) *^b^*

Abbreviations: ATM, adipose tissue mass; BCM, body cell mass; BMI, body mass index; CSA, cross–sectional area; FTI, fat tissue index; FTM, fat tissue mass; HGS, handgrip strength; IQR, interquartile range; LTI, lean tissue index; LTM, lean tissue mass; MAC, mid-arm circumference; MAMA, mid-arm muscle area, MAMC, mid-arm muscle circumference; MID, mid-point; PEW, protein energy wasting; RF, *rectus femoris*; TSF, triceps skinfold; US, ultrasound; VI, *vastus intermedius*. *^a^* Data expressed as the median (IQR); analyzed using the Mann−Whitney U-test. *^b^* Significance at *p* < 0.001. *^c^* Significance at *p* < 0.05.

**Table 3:** ROC analysis of US, BIS, and HGS for PEW risk.

Parameters	AUC	95% CI	*p*-Value	Sensitivity	Specificity
RF_MID_ (cm)	0.639	0.57–0.71	<0.001	0.531	0.748
VI_MID_ (cm)	0.702	0.64–0.77	<0.001	0.827	0.474
RF_2/3_ (cm)	0.647	0.58–0.72	<0.001	0.593	0.663
VI_2/3_ (cm)	0.696	0.63–0.76	<0.001	0.654	0.656
RF_CSA_ (cm^2^)	0.686	0.62–0.75	<0.001	0.728	0.556
BIS–LTM	0.515	0.45–0.58	0.693	0.797	0.300
BIS–LTI	0.581	0.51–0.65	0.029	0.658	0.539
BIS–BCM	0.545	0.48–0.61	0.229	0.772	0.378
HGS	0.532	0.46–0.60	0.380	0.620	0.491

Abbreviations: AUC, area under the curve; BCM, body cell mass; BIS, bio-impedance spectroscopy; CI, confidence interval; CSA, cross-sectional area; HGS, handgrip strength; LTI, lean tissue index; LTM, lean tissue mass; MID, mid-point; PEW, protein energy wasting; RF, *rectus femoris*; ROC, receiver operating characteristic; US, ultrasound; VI, *vastus intermedius*.

**Table 4:** ROC analysis for US measures for determination of PEW risk.

US Measures	Men			Women		
	AUC	95% CI	*p*-Value	AUC	95% CI	*p*-Value
RF_MID_ (cm)	0.68	0.60–0.77	<0.001	0.75	0.64–0.85	<0.001
VI_MID_ (cm)	0.71	0.63–0.79	<0.001	0.69	0.58–0.80	0.004
RF_2/3_ (cm)	0.67	0.59–0.76	<0.001	0.72	0.60–0.83	0.001
VI_2/3_ (cm)	0.70	0.62–0.79	<0.001	0.70	0.58–0.81	0.003
RF_CSA_ (cm^2^)	0.74	0.66–0.82	<0.001	0.80	0.70–0.90	<0.001

Abbreviations: AUC, area under the curve; CI, confidence interval; CSA, cross-sectional area; MID, mid-point; PEW, protein energy wasting; RF, *rectus femoris*; ROC, receiver operating characteristic; US, ultrasound; VI, *vastus intermedius*.

**Table 5:** Sensitivity and specificity of the RF_CSA_ in discriminating PEW risk.

	Men (<6.00 cm^2^)	Women (<4.47 cm^2^)
Sensitivity	0.618	0.769
Specificity	0.797	0.803

Abbreviations: CSA, cross-sectional area; PEW, protein energy wasting; RF, *rectus femoris*.

**Table 6:** Association of the RF_CSA_ gender-specific values with PEW risk.

PEW Risk	Odds Ratio	95% CI	*p*-Value
*Unadjusted*			
Low RF_CSA_	8.00	4.62–13.86	<0.001
High RF_CSA_	Reference		
*Adjusted ^a^*			
Low RF_CSA_	8.63	4.80–15.50	<0.001
High RF_CSA_	Reference		

Abbreviations: CI, confidence interval; CSA, cross-sectional area; PEW; protein energy wasting; RF, *rectus femoris*. Note: RF_CSA_ gender-specific values for the PEW risk was < 6.00 cm^2^ for men and < 4.47 cm^2^ for women. *^a^* Data was adjusted for age, ethnicity, dialysis vintage, and comorbidities.

## Abbreviations

AOR	Adjusted odds ratio
ATM	Adipose tissue mass
AUC	Area under the curve
BCM	Body cell mass
BIA	Bio-impedance analysis
BIS	Bio-impedance spectroscopy
BMI	Body mass index
CI	Confidence interval
CKD	Chronic kidney disease
CSA	Cross-sectional area
CT	Computed tomography
DEI	Dietary energy intake
FTI	Fat tissue index
FTM	Fat tissue mass
HD	Hemodialysis
HGS	Handgrip strength
hsCRP	High-sensitivity C-reactive protein
IBW	Ideal body weight
IL-6	Interleukin-6
IQR	Interquartile range
ISAK	International Society for the Advancement of Kinanthropometry
ISRNM	International Society of Renal Nutrition and Metabolism
LBM	Lean body mass
LTI	Lean tissue index
LTM	Lean tissue mass
MAC	Mid-arm circumference
MAMA	Mid-arm muscle area
MAMC	Mid-arm muscle circumference
MIS	Malnutrition–inflammation score
PATCH	Palm Tocotrienol in Chronic Hemodialysis
PEW	Protein energy wasting
QMT	Quadriceps muscle thickness
RF	*Rectus femoris*
ROC	Receiver operating characteristic
SD	Standard deviation
TSF	Triceps skinfold
US	Ultrasound
VI	*Vastus intermedius*

## References

[1] Fouque D., Kalantar-Zadeh K., Kopple J.D., Cano N., Chauveau P., Cuppari L., A Franch H., Guarnieri G.L., Ikizler T., A Kaysen G. (2008). A proposed nomenclature and diagnostic criteria for protein–energy wasting in acute and chronic kidney disease. Kidney Int..

[2] Carrero J.J., Thomas F., Nagy K., Arogundade F., Avesani C.M., Chan M., Chmielewski M., Cordeiro A.C., Espinosa-Cuevas A., Fiaccadori E. (2018). Global Prevalence of Protein-Energy Wasting in Kidney Disease: A Meta-analysis of Contemporary Observational Studies From the International Society of Renal Nutrition and Metabolism. J. Ren. Nutr..

[3] Mourtzakis M., Wischmeyer P. (2014). Bedside ultrasound measurement of skeletal muscle. Curr. Opin. Clin. Nutr. Metab. Care.

[4] Workeneh B.T., E Mitch W. (2010). Review of muscle wasting associated with chronic kidney disease. Am. J. Clin. Nutr..

[5] Carrero J.J., Johansen K.L., Lindholm B., Stenvinkel P., Cuppari L., Avesani C.M. (2016). Screening for muscle wasting and dysfunction in patients with chronic kidney disease. Kidney Int..

[6] Norton K., Eston R. (2018). Kinanthropometry and Exercise Physiology.

[7] Khalil S.F., Mohktar M.S., Ibrahim F. (2014). The Theory and Fundamentals of Bioimpedance Analysis in Clinical Status Monitoring and Diagnosis of Diseases. Sensors.

[8] Chamney P.W., Wabel P., Moissl U.M., Müller M.J., Bosy-Westphal A., Korth O., Fuller N.J. (2007). A whole-body model to distinguish excess fluid from the hydration of major body tissues. Am. J. Clin. Nutr..

[9] Sahathevan S., Khor B., Yeong C.H., Tan T.H., Mohaideen A.K.M., Ng H.M., Ong G.R., Narayanan S.S., Gafor A.H.A., Goh B.L. (2020). Validity of Ultrasound Imaging in Measuring Quadriceps Muscle Thickness and Cross-Sectional Area in Patients Receiving Maintenance Hemodialysis. J. Parenter. Enter. Nutr..

[10] Martín C.A.G., Zepeda E.M., Méndez O.A.L. (2017). Bedside Ultrasound Measurement of Rectus Femoris: A Tutorial for the Nutrition Support Clinician. J. Nutr. Metab..

[11] Sabatino A., Regolisti G., Delsante M., Di Motta T., Cantarelli C., Pioli S., Grassi G., Batini V., Gregorini M., Fiaccadori E. (2019). Noninvasive evaluation of muscle mass by ultrasonography of quadriceps femoris muscle in End-Stage Renal Disease patients on hemodialysis. Clin. Nutr..

[12] Scarborough D.M., Krebs D.E., Harris B.A. (1999). Quadriceps muscle strength and dynamic stability in elderly persons. Gait Posture.

[13] Gruther W., Benesch T., Zorn C., Paternostro-Sluga T., Quittan M., Fialka-Moser V., Spiss C., Kainberger F., Crevenna R. (2008). Muscle wasting in intensive care patients: Ultrasound observation of the M. quadriceps femoris muscle layer. Acta Derm. Venereol..

[14] Menon M.K., Houchen L., Harrison S., Singh S.J., Morgan M.D., Steiner M.C. (2012). Ultrasound assessment of lower limb muscle mass in response to resistance training in COPD. Respir. Res..

[15] Heymsfield S.B., McManus C., Smith J., Stevens V., Nixon D.W. (1982). Anthropometric measurement of muscle mass: Revised equations for calculating bone-free arm muscle area. Am. J. Clin. Nutr..

[16] Ikizler T.A., Burrowes J.D., Byham-Gray L.D., Campbell K.L., Carrero J.-J., Chan W., Fouque D., Friedman A.N., Ghaddar S., Goldstein-Fuchs D.J. (2020). KDOQI Clinical Practice Guideline for Nutrition in CKD: 2020 Update. Am. J. Kidney Dis..

[17] Kalantar-Zadeh K., Ikizler T., Block G., Avram M.M., Kopple J.D. (2003). Malnutrition-inflammation complex syndrome in dialysis patients: Causes and consequences. Am. J. Kidney Dis..

[18] Garagarza C., Flores A.L., Valente A. (2017). Influence of Body Composition and Nutrition Parameters in Handgrip Strength: Are There Differences by Sex in Hemodialysis Patients?. Nutr. Clin. Pr..

[19] Toledo F.R.U., Antunes A.A.U., Vannini F.C.D.U., Silveira L.V.A.U., Martin L.C.U., Barretti P., Caramori J.C.T. (2013). Validity of malnutrition scores for predicting mortality in chronic hemodialysis patients. Int. Urol. Nephrol..

[20] Isoyama N., Qureshi A.R., Avesani C.M., Lindholm B., Bàràny P., Heimbürger O., Cederholm T., Stenvinkel P., Carrero J.J. (2014). Comparative Associations of Muscle Mass and Muscle Strength with Mortality in Dialysis Patients. Clin. J. Am. Soc. Nephrol..

[21] Kirchengast S. (2010). Gender Differences in Body Composition from Childhood to Old Age: An Evolutionary Point of View. J. Life Sci..

[22] De Souza V.A., Oliveira D., Cupolilo E.N., Miranda C.S., Colugnati F.A.B., Mansur H.N., Fernandes N.M.D.S., Bastos M.G. (2018). Rectus femoris muscle mass evaluation by ultrasound: Facilitating sarcopenia diagnosis in pre-dialysis chronic kidney disease stages. Clinics.

[23] Studenski S.A., Peters K.W., Alley D.E., Cawthon P.M., McLean R.R., Harris T.B., Ferrucci L., Guralnik J.M., Fragala M.S., Kenny A.M. (2014). The FNIH Sarcopenia Project: Rationale, Study Description, Conference Recommendations, and Final Estimates. J. Gerontol. Ser. A. Boil. Sci. Med. Sci..

[24] Muscaritoli M., Molfino A., Bollea M.R., Fanelli F.R. (2009). Malnutrition and wasting in renal disease. Curr. Opin. Clin. Nutr. Metab. Care.

[25] Chen L.-K., Lee W.-J., Peng L.-N., Liu L.-K., Arai H., Akishita M. (2016). Recent Advances in Sarcopenia Research in Asia: 2016 Update From the Asian Working Group for Sarcopenia. J. Am. Med. Dir. Assoc..

[26] Wilkinson T.J., Gould D.W., Nixon D.G.D., Watson E.L., Smith A. (2019). Quality over quantity? Association of skeletal muscle myosteatosis and myofibrosis on physical function in chronic kidney disease. Nephrol. Dial. Transpl..

[27] Seymour J.M., Ward K., Sidhu P.S., Puthucheary Z., Steier J., Jolley C.J., Rafferty G.F., Polkey M.I., Moxham J. (2009). Ultrasound measurement of rectus femoris cross-sectional area and the relationship with quadriceps strength in COPD. Thorax.

[28] Trappe T.A., Lindquist D.M., Carrithers J.A. (2001). Muscle-specific atrophy of the quadriceps femoris with aging. J. Appl. Physiol..

[29] Mueller N., Murthy S., Tainter C.R., Lee J., Riddell K., Fintelmann F.J., Grabitz S.D., Timm F.P., Levi B., Kurth T. (2016). Can Sarcopenia Quantified by Ultrasound of the Rectus Femoris Muscle Predict Adverse Outcome of Surgical Intensive Care Unit Patients as well as Frailty? A Prospective, Observational Cohort Study. Ann. Surg..

[30] Battaglia Y., Ullo I., Massarenti S., Esposito P., Prencipe M.A., Ciancio G., Provenzano M., Fiorini F., Andreucci M., Storari A. (2020). Ultrasonography of Quadriceps Femoris Muscle and Subcutaneous Fat Tissue and Body Composition by BIVA in Chronic Dialysis Patients. Nutrients.

[31] Bury C., DeChicco R., Nowak D., Lopez R., He L., Jacob S., Kirby D.F., Rahman N., Cresci G. (2020). Use of Bedside Ultrasound to Assess Muscle Changes in the Critically Ill Surgical Patient. J. Parenter. Enter. Nutr..

[32] Gould D.W., Watson E.L., Wilkinson T.J., Wormleighton J., Xenophontos S., Viana J.L., Smith A. (2019). Ultrasound assessment of muscle mass in response to exercise training in chronic kidney disease: A comparison with MRI. J. Cachexia Sarcopeni..

[33] Yamada M., Kimura Y., Ishiyama D., Nishio N., Otobe Y., Tanaka T., Ohji S., Koyama S., Sato A., Suzuki M. (2019). Synergistic effect of bodyweight resistance exercise and protein supplementation on skeletal muscle in sarcopenic or dynapenic older adults. Geriatr. Gerontol. Int..

[34] Sabatino A., Regolisti G., Bozzoli L., Fani F., Antoniotti R., Maggiore U., Fiaccadori E. (2017). Reliability of bedside ultrasound for measurement of quadriceps muscle thickness in critically ill patients with acute kidney injury. Clin. Nutr..

